# Towards a shared mental model of progressive competence in postgraduate medical education

**Published:** 2018-07-27

**Authors:** Eric Prost

**Affiliations:** 1 Department of Psychiatry, Queen’s University, Ontario, Canada

## Abstract

Many professions have hierarchies and a promotion structure. Postgraduate medicine has a tradition of promoting residents based on time spent in a certain specialty. The military, too, may promote its personnel based on factors other than just merit. Both professions have been criticized for divorcing competence from promotion. While Competency-Based Medical Education (CBME) partly solves this problem in medicine, many models of CBME, including the Canadian one, retain distinct stages of training. We urgently need a shared mental model of what a learner in each stage looks like. Some models have been proposed but fall short.

Many institutions, from the military to medicine to the Catholic Church to a construction site, have hierarchies. They make sense. And, often, the higher the stakes are, the more rigid the hierarchy. In the army, the chain of command is paramount: lower ranks of officers have some permission to make their own decisions on the ground and in the moment as the situation unfolds; however, orders from above, especially if they are explicit, are not to be ignored.

In medicine, the situation is similar. The chain of command might instead be construed as the chain of authority and responsibility. From medical student to junior resident to senior resident to attending physician, the hierarchy has a purpose and a logic. At each level, the practitioner has certain tasks and reports to the person above, both for permission and for help. The lower ranks have considerable leeway, though, to exercise their own judgment, as well as considerable authority to effect change through performing procedures, ordering investigations, and prescribing medicines without needing to check with those of higher rank. This is especially true once the learner possesses an MD degree and is licensed, thereby officially needing no one to co-sign orders. There is considerable opportunity for individuals to both help and harm.

The medical profession is one where responsibility and authority are often given early and in large quantities - and, I think, with minimal oversight or assurance that the practitioner possesses the required skills. And, historically, transitions to higher stages of responsibility and authority have been based on little objective data.

Consider promotion in rank in the U.S. military. While the uniformed services have differences—army, navy, air force, marine corps—there are constants. To rise from the lowest levels of enlistment, the criteria are mostly time-based. If a private has spent a certain number of months in his or her initial rank, he or she may be eligible for promotion as long as there is also an available spot in the new rank or pay grade. These decisions can often be made at the “local” level by those who know the soldier and are his or her superiors within the unit.

At a higher level of promotion, a point system is used. This may include assigning points for passing certain examinations, having good marksmanship, being decorated, or having civilian degrees or physical fitness. At the highest levels, the promotion is yet more centralized and is the responsibility of a board that reviews the soldier’s career and may even have him or her present in person before the board.^1^

Yet despite the rigour of this process and the increasing centralization and structure of the process as the rank (and, I assume, the stakes) increases, the whole system has been criticized. In one study of West Point graduates, only 30% believed that the military “does a good job of promoting the right officers to General.”^2^ Despite the point system and the promotion structure, some officers sense that they are promoted after certain predictable lengths of service rather than on merit. This, in turn, may have effects on retention of the best and brightest in military careers. If the creative or the independent or the smartest officers do not get promoted correctly and efficiently, they will leave to become civilian CEOs, so the argument goes. When promotion and competence do not align, there are problems.

This, too, has long been the problem in medicine: residents are promoted to the next post-graduate year after a certain number of months on each rotation and after a year at each level of training. The less competent in medicine are sometimes promoted despite being unsafe and unprofessional. If competence in the military is ignored during promotion, competence in medicine isn’t even measured. We give authority and responsibility without knowing for sure if it is presaged by competence. We must remarry competence and promotion in medicine.

This brings us to Competency-Based Medical Education (CBME). Within this new model, residents are promoted by getting discrete professional activities entrusted to them. These discrete tasks are called Entrustable Professional Activities (EPAs). For example, a basic professional activity of most medical specialties is that of taking an accurate history from an ill patient and then generating a plausible differential diagnosis. The CBME model of postgraduate medical education ensures that promotion follows competence. In fact, competence equals promotion. The fulfillment of each Entrustable Professional Activity - repeating it under observation multiple times until it is consistently performed competently - is, effectively, the promotion structure. If a resident is competent at an EPA, she is promoted by being told that the EPA is entrusted to her. The EPA is signed off. That’s the promotion.

Where Competency-Based Medical Education is inconsistent with its philosophy, however, is that it still retains stages, ensuring that a resident promotion structure exists in addition to the task of accomplishing EPAs. The Royal College of Physicians and Surgeons of Canada has established stages for all residency-training programs within CBME.^3^ The main four are these: (1) Transition to Discipline; (2) Foundations of Discipline; (3) Core of Discipline; and (4) Transition to Practice. Each stage contains multiple EPAs. This has face value because, as I’ve mentioned, we are stage-based creatures, whether as bishops and archbishops, captains and colonels, journeymen and masters, or junior (foundations) residents and senior (core) residents. However, these stages are inconsistent with the spirit of the whole CBME endeavour.

Within CBME, promotion itself should be seen as simply the act of entrustment of each EPA. Instead, however, we have retained the hierarchy and thus relegated the entrustment of professional activities to something akin to receiving medals within the military. Each entrusted activity - each decoration - becomes just a piece of the bigger goal of promotion to the next stage, which, in turn, is likely where residents will focus their energies. This is not all a bad thing, since perhaps EPAs will be seen as important smaller goals while stage promotion will be seen as the greater longer-term marker of progress.

Either way, since the stages seem here to stay, we must make the most of them. Various efforts are ongoing to add flesh to the stages, to render them more concrete or understandable, rather than to see them just as collections of EPAs, just receptacles for discordant entrustable activities.

The *RIME* model is one attempt.^4^ Originally designed to correspond to the four years of medical school, this model attempts to create a shared mental model of what a medical student in general should be able to do during each year. He should first be a *R*ecorder of facts he collects from patients, charts, and the medical literature; then an *I*nterpreter of the data, able to draw conclusions through clinical reasoning; then a *M*anager of patients, able to design and implement management plans for symptoms and diagnoses; and, finally, an *E*ducator, one who can distill information into logical chunks and pass on knowledge to others, whether to junior learners or patients and their families. This model is elegant and has face value. Unfortunately, it is not as applicable to postgraduate medicine as it is to undergraduate: residents must not wait until their third stage or year, for example, to begin to manage patients. We need a mental model suitable for postgraduate education.

Residency is essentially an apprenticeship model. Attempts by educational theorists to call this type of learning by other names such as “peripheral participation” or “situational learning” seem to me to be putting a hat on a horse. Apprenticeship models have existed for centuries and in many cultures.^5^ Let’s call an apprenticeship what it is, and not hide its nature even from ourselves.

The main goal of having an apprenticeship model is to allow the learner to be involved (and learn) with varying (and, hopefully, increasing) levels of responsibility, from observing, to doing some parts of a larger task, to performing more of the task, to performing all of it under supervision, etc. This may take years, as it did for medieval artisans who went from apprentices to journeymen to masters. Often, in this model, the transition from one stage to the next requires an objective measure such as creating a “masterpiece” to become a master in, for example, a carpenters’ guild.

Should we see the four stages of CBME as the four stages of a traditional apprenticeship? After all, why create a new shared mental model when, perhaps, we already have one? Stage One could be the Pre-apprenticeship stage (Transition to Discipline) when the new recruit to the specific medical specialty learns the ropes and performs tasks that are menial but important, spending time with supervisors who will not be her ultimate mentors but who, in this stage, have a lot to offer (i.e., “off service” supervisors). Stage Two is the Apprenticeship stage (Foundations of Discipline) where the novice is a true apprentice - observing often, doing parts of tasks, and focusing on the basic but fundamental parts of the calling. Stage Three is the Journeyman stage (Core) where the learners perform often, observe sometimes, and focus on more specialized tasks. Like the carpentry or plumbing journeyman, they “do, do, do.” Finally, the Master stage is where observation is seldom, the bar is high (that of a “master,” after all), and the learner is almost released (though always willing to learn).

As we are already implementing Competency-Based Medical Education in Canada, we urgently need a shared mental model of what each stage looks like. The Entrustable Professional Activities themselves, if written well, are concrete and tangible. Our faculty members and residents can understand what is expected within each of these tasks. The stages that we have retained in Canadian postgraduate medical education, however, need re-defining.

What does a “Core” resident look like - if she needs to look like more than just someone who can be trusted with a certain bag of EPAs? Does she look like a Journeyman in a medieval guild? Does he look like a “manager” in third year medical school? Hopefully, he doesn’t look like a Brigadier General who has been promoted based on years of service and just following rules. But I do hope she’s safe, professional, and competent.

What is most important is that we all see them in roughly the same way: we need a shared mental model.
